# Weekend Headaches in School-Age Children

**DOI:** 10.3390/healthcare12010060

**Published:** 2023-12-26

**Authors:** Daniel Triadou, Yoel Bar-Shalom, Mordechai Pollak, Shoshana Gal, Keren Nathan, Megi Yakovlev, Jacob Genizi

**Affiliations:** 1 Pediatric Department, Bnai Zion Medical Center, Haifa 3104802, Israel; daniel.triadou@b-zion.org.il (D.T.); yoel.bar-shalom@b-zion.org.il (Y.B.-S.); shoshana.gal@b-zion.org.il (S.G.); keren.nathan@b-zion.org.il (K.N.); megi.yakovlev01@upol.cz (M.Y.); jacob.genizi@b-zion.org.il (J.G.); 2 Bruce Rappaport Faulty of Medicine, Technion, Haifa 3109601, Israel

**Keywords:** headache, triggers, weekend headache, learning difficulties

## Abstract

Children commonly encounter primary headaches, with various factors playing a role in their onset. The daily routine notably contributes to the occurrence of primary headaches in children. This study aims to profile children experiencing headaches on weekends (WH) in comparison to those primarily having headaches midweek (MWH). Out of 109 children visiting a pediatric headache clinic, 60 prospectively filled out questionnaires regarding their headaches. The average age was 11.8 years, and 63% were of female sex. Most of the children suffered from migraine headaches (60%), while the rest suffered from tension-type headaches (TTH, 15%), mixed headaches (17%), or undetermined headaches (8%). None of the children suffered from a headache only on the weekend. In contrast, 14 (23%) children suffered from a headache exclusively in midweek. Children with learning difficulties were similarly distributed between the WH and the MWH groups (48% and 52%, respectively). Children without learning difficulties suffered significantly more from MWH compared to WH (79% vs. 21%, respectively). In conclusion, children did not suffer from WH alone. Self-reported triggers were not significantly different in WH and MWH patients. Proper profiling of headache types and triggers may lead to more accurate management of these patients.

## 1. Introduction

Headache is one of the most common complaints in children and adolescents. It is recognized as one of the top medical and neurologic contributors to the global burden of disease and is a leading cause of disability in adolescents and young adults [1]. Recurrent, severe headaches are common in children. In the United States, approximately 20 percent of children aged 4 to 18 report having had notable recurrent headaches in the past 12 months, and the prevalence tends to increase with age [2]. The International Classification of Headache Disorders, 3rd edition (ICHD-3), provides detailed diagnostic criteria for primary headaches, which include mainly migraine and tension-type headaches (TTH) [3]. A diary in which the quality, location, severity, timing, precipitating and palliating factors, and associated features of the headache are recorded prospectively, not subject to recall error, may be a useful adjunct if the child is willing and able to complete it daily [4]. Headaches can be triggered by various factors, including academic or emotional stress, lack of sleep or exercise, multiple screen hours, untidy meals, etc. It can be assumed that the occurrence of these triggers may differ between weekends and regular school days, consequently impacting the frequency of headaches [5,6,7,8]. There are a few studies that investigated weekend headaches among the adult population [9,10,11], and to our knowledge, only two studies mentioned differences in headache patterns between days of the week among school-aged children [12,13]. Mehta et al. [12] reported the rate of headaches on weekends to be 5.9%, compared to 28.5% on Monday, among children 7–14 years of age. On the other hand, Larsson et al. [13] did not find a significant difference in headache frequencies between weekdays and weekends, although the peak incidence was on Thursday. Finally, learning difficulties are more common in children and adolescents who suffer from primary headaches [14], and since much of the headache in children is attributed to school, we hypothesized that the distribution of headaches along the week might be different in children with learning difficulties compared to those without. 

The main goal of this study was to assess the presence of weekend headaches in school-age children. Our study hypothesis was that children, unlike adults, do not suffer from weekend headaches. Children’s resilient physiology and generally lower stress levels contribute to a consistent headache frequency, unaffected by weekends. Unlike adults, variations in children’s routines during weekends may not significantly impact their well-being. Their adaptability to changes in sleep, diet, and activities is likely to mitigate the prevalence of headaches during weekends. In addition, we aimed to characterize the children who suffer from weekend headaches (WH) compared to those that suffer more midweek headaches (MWH) and to define the different triggers between those two groups. At last, we aimed to identify possible connections between learning difficulties and the appearance of MWH.

## 2. Patients and Methods

This is a prospective follow-up study focused on observing children aged 6–17 years who experience primary headaches and receive treatment at the pediatric neurology clinic located at Bnai Zion Medical Center, Haifa, Israel. The recruitment of participants for this study occurred during the period spanning February 2022 to September 2023. All participants received a paper headache diary, which they filled out for at least 4 weeks. This period has been found to be optimal regarding compliance, validity, and reliability of ratings [15]. Patients were directed to record the date of onset of the headache, its severity, duration, and the time of occurrence throughout the day. Furthermore, patients were asked to relate to potential triggers, which included factors like sleep duration, exercise timing, screen exposure hours in terms of average, higher, or lower than usual, and their subjective perception of academic or emotional stress. The clinic team took proactive measures to ensure that the questionnaire was consistently and accurately completed. Additional data regarding triggers for the headache as well as information regarding learning difficulties were obtained. A comparative analysis of the demographic and clinical profiles, as well as the triggers for the patients, was conducted. As well, patients and caregivers were asked about the presence of learning difficulties, and a subgroup of patients with learning difficulties was formed accordingly. We did not include exclusively formally diagnosed children with learning disorders or ADHD; rather, we included patients whose caregivers reported having learning challenges. We chose this strategy since we did not look for a pathophysiological connection between headaches and learning disorders; rather, we wanted to assess the difference between schooldays and weekends and the relation to any learning challenges. We then assessed headache patterns in this particular group over the course of the week. In Israel, Friday and Saturday are considered weekend days, while Sunday through Thursday are regular weekdays. Despite that, some children attend school six days a week, including Friday. Therefore, in order to achieve more accurate results, we categorized Sunday through Thursday as the middle of the week while designating Saturday exclusively as the weekend. 

For the statistical analysis, the data were processed and analyzed using SPSS software. X^2^ tests were performed for categorical data. Odds ratios with 95% confidence intervals were computed, and statistical significance was considered as *p* < 0.05. The study was approved by the hospital’s ethics committee (IRB # Materials BNZ-0138-21).

## 3. Results

A total of 109 children were enrolled in this study. Among them, 60 children (56%) diligently completed a comprehensive headache diary, while the remaining 49 children (44%) were not included in the final analysis. Of those, 34/49 were excluded due to inadequate compliance and 15/49 due to the resolution of their symptoms. The majority of the children suffered from migraine (60%), while the rest suffered from TTH (15%), mixed headaches (17%), or undetermined headaches (8%). Thirteen children (22%) met the definition of chronic headache; of those, seven had migraine characteristics, three had TTH characteristics, two had mixed characteristics, and one had undetermined characteristics. The average age was 11.8 years, with most of the children (55%) attending elementary school (Table 1). Most of the diaries were completed over a period of 6 weeks, with an average of 5.7 weeks. A total of 815 events of headache were recorded over a cumulative period of 338 weeks. There was a wide range of headache frequencies among each child individually, ranging from 1 to 36 events over the course of the study. The weekly average of headache events was 2.41 per child during the study period.

The vast majority of children suffered from headaches both on weekend and midweek days. Therefore, children with more WH than MWH were included in the WH group, despite also having midweek headaches. Similarly, those with mostly mid-week headaches were included in the MWH group. A similar approach was used in a previous study in adults [11]. Of the 60 children who were included in the study, 40 (67%) suffered predominantly midweek headaches, while 20 (33%) suffered mainly on the weekends. None of the children suffered from a headache only on the weekend, but 14 (23%) children suffered from a headache exclusively on midweek days.

The demographic data according to the WH and MWH groups can be seen in Table 1. There was no significant difference in terms of age and gender between children with WH and children with MWH.

The distribution of different triggers for headaches in patients with WH and MWH can be seen in Table 2. There was no significant difference in the distribution of triggers between the children in both groups. 

The children were further categorized according to their specific headache type. For all headache subtypes, there was no significant difference between those with WH and MWH (Table 3). As well, we did not find a significant difference between the two groups when comparing the frequency of chronic headaches. 

Interestingly, a significantly higher proportion of children with WH compared to MWH reported to have learning difficulties, such as learning disabilities or ADHD (65% vs. 35%, respectively, *p* < 0.03). When isolating the subgroup of 14 children who suffered from MWH exclusively, the proportion of children reporting learning difficulties was even lower (29%).

The distribution of headaches during the week in children with primary headaches and learning difficulties compared with children without learning difficulties can be seen in Table 4. A relatively high percentage of children with primary headaches were reported to have learning difficulties (45%).

## 4. Discussion

To our knowledge, this is the first prospective study examining MWH versus WH in children with primary headaches. Our main findings were that children do not suffer from WH exclusively and that children with learning difficulties tend to have more WH compared with those without learning difficulties. 

In a previous study of schoolchildren with primary headaches, estimates of headache frequency were higher in the prospective diary compared to questionnaires and interviews [4,16], and so we preferred to adopt this method. Another important advantage was that the average duration of filling in a diary was about 6 weeks, not just 3 weeks, which was described as sufficient to produce reliable information considering compliance [15]. At last, the response rate was high and was composed of a wide variety of children of different ages, different regions, and different cultures, therefore giving a comprehensive picture. In our study, we included in the WH group the children whose headaches appear more frequently during the weekends, compared to their appearance during the middle of the week, as studied by Torelli P et al. in adults [11]. 

As opposed to adults [9,10,11], our findings suggest that children tend to have more headaches during the week and never only on the weekends. In adults, Saturday was the most common day for migraines. The difference between adults and children may be partly due to differences in the triggers for the headaches. Excessive alcohol and coffee consumption, irregular sleep hours, and family stress are all typical weekend triggers that are more prevalent among adults and less present in children [9]. 

We found a trend towards a higher prevalence of MWH compared with WH. Although there are very few studies in children that refer to weekend headaches, a study from India reported a higher incidence of headache after the weekend among children aged 7–14 years [12]. Another study did not find a significant difference in headache incidence between weekdays and weekends in school-aged children, although the peak incidence was in the middle of the week, mostly on Thursday, and less on the weekend [13]. In both studies, headaches from the entire school population were examined, and a specific characterization of those with recurrent headaches was not performed. Overall, our study did not reveal a significant difference in the prevalence of headaches between mid-week and weekends. One possible explanation for this finding could be the lack of substantial differences in headache triggers such as the duration of screen time and physical activities between mid-week and weekends.

There was no significant difference in the mean ages between the two groups (Table 1). However, in the WH group, there were relatively more children in elementary school, maybe since they experienced less academic pressure. Importantly, some of the teenagers filled in the diaries themselves, while young children had their diaries filled in by their parents. It is possible that headaches occurring at school would not be documented by the younger children since parents are not present with their children during school hours. In addition, the parents are also busier during the week and spend less time with their children, which may lead to underreporting. Therefore, it is possible that, among small children, midweek headaches might be underestimated.

Among adolescents in junior high and high school, there was more MWH, possibly attributed to academic stress, which increases with age. There is more pressure to achieve and less tolerance for failure. Adolescents may also be more manipulative than small children and complain of a headache for secondary gain. Chronic or recurrent pain may be associated with anxiety, depression, and behavior problems, which become more important as the child enters adolescence. Moreover, in the group of adolescents, there are other habits that might trigger the appearance of a primary headache, such as the consumption of coffee, alcohol, cigarettes, etc., that were not examined in our study. The use of these substances or withdrawal from them may cause headaches, as can be seen in adults [8,11,17,18]. A single study from Norway showed that among the teenagers, headaches appeared more in the afternoon, which they propose to be related to the accumulation of the academic load throughout the day and throughout the week [13]. 

Before puberty, the prevalence of headaches among boys and girls is similar [2], but after this age, a significant increase in the frequency of headaches can be seen in girls [19]. In a previous study that was performed on adults suffering from tension-type headaches, there was a rise in attacks during weekends [10]. Another study reported that weekend headaches typically affected men more than women [11]. In our study, however, WH were not more common among males (45% vs. 55%, *p* = 0.34). This might be attributed to the differences in triggers between children and adults. 

We requested our patients to outline specific triggers, such as academic stress, emotional stress, or daily habits, and subsequently compared these factors between the two groups (Table 2). It should be noted that each child may have several different triggers for the appearance of a headache during the study period, and they were not limited to finding a single trigger. Although we did not find any statistically significant difference between the groups, academic stress was reported more commonly among the group of children with MWH (48% vs. 35%) and was reported less in the WH group. Since the children do not go to school over the weekend, it is easy to understand this difference. However, it should be noted that those with WH also reported academic stress. In adults, occupational stress, which can correspond to academic stress in children, is assumed to be one of the main triggers for migraine attacks during the week [20]. Previous studies reported academic stress as a leading factor in headaches in children [12,19]. However, in our study, academic stress was reported to be the least common headache trigger, especially during the weekends. As opposed to these findings, older studies showed that academic stress is a significant trigger for headaches in children [21,22,23]. Possibly, nowadays other triggers, such as emotional stress, take higher importance as a trigger for headaches in accordance with the change that has occurred in the population in the modern world. If so, the relationship between learning difficulties and headaches during midweek might be similarly influenced.

Emotional stress is a well-known trigger for primary headaches. Children with migraine seem to have more emotional symptoms than children without migraine and more associated somatic complaints [12,19,24]. Emotional stress likely arises for various reasons throughout the week, both at school and after school, as well as at home during the weekends. On one hand, weekends offer more free time, providing space for concerns that could elevate the risk of headaches. It is plausible that the origins of emotional stress stem from home or family, which becomes more pronounced on weekends. On the other hand, during school, social and academic pressures can be the source of emotional stress. These multiple factors contribute to the absence of a significant difference between weekend headaches and midweek headaches in the context of emotional stress.

Overall, in children who suffered from WH, lifestyle triggers such as lack of sleep, excess screen hours, and lack of physical activity were prominent but not significantly increased compared to the MWH. Gariépy et al. showed that a late timing of sleep was associated with more headaches in adolescents, and on weekends, the average bedtime was later [6]. Importantly, although bedtime is usually later on the weekend, the duration of sleep is not necessarily shorter, and often even the opposite. It is therefore not surprising that we did not find a significant difference in terms of lack of sleep reported between the WH and MWH. 

In terms of screen time, no significant difference was found between MWH and WH. Headaches were found to be more prevalent among children with longer television watching time [7]. This falls in line with the results presented in this current study, which show a high incidence of headaches that are triggered by excess screen time. The fact that today children of all ages spend many hours in front of a screen, both during school days and at the weekends, contributed to the fact that no significant difference was found between the two groups. It has been shown that individuals with migraines report lower levels of physical activity compared to nonheadache controls, even on days in which headaches were not experienced [5]. Most of the children in this study suffered from migraines, and it is therefore not surprising that a lack of physical activity was reported often, both in the middle of the week and at weekends. In adults, the effects of other habits, such as caffeine consumption, were also found to trigger headaches at the end of the week compared to the middle of the week [8,17,18]. One study showed that weekend headaches are linked to caffeine withdrawal [18], while in another study, no changes were found in the intake of substances such as coffee and alcohol or in cigarette smoking over the different days of the week [11]. Consumption or withdrawal of substances such as caffeine, alcohol, and nicotine could have affected the adolescent population in this study as well.

The prevalence of migraine-type headaches was similar in the WH and MWH groups (Table 3). In a previous study in adults, Torelli et al. proposed that the weekend may be a triggering factor in migraine attacks and play a major role in episodic tension-type headaches [10]. In our sample, a trend toward higher proportions of chronic headaches was seen in MWH compared to WH. This was not statistically significant, possibly due to the relatively small number of patients with chronic headaches. Individuals dealing with chronic headaches endure almost daily discomfort and are not significantly impacted on the weekends. A study in adults showed that apart from certain features that appear to be particular to weekend headaches, such as increased pain intensity, weekend headaches typically fulfill the diagnostic criteria of the primary headaches from which they evolve [10]. In another study, the intensity of headaches was positively related to frequency [13]. In our study, the intensity and duration of the headache attacks were not documented accurately and therefore not considered for the analysis. Despite this limitation, the goal of this study was to differentiate between children that suffer from weekend headaches compared with weekday headaches and not between the attacks themselves.

Children with WH had more learning difficulties than children with MWH. Previous studies, including our previous study, showed the connection between headaches and learning difficulties and that much of the headache in children is attributed to school [21,25]. We found that 24% were formerly diagnosed with learning disabilities, and 28% were diagnosed with attention deficit disorder [14]. Mathematics or science test dates and post-weekend days in school were found to increase the occurrence of headaches in school-attending children [12]. In addition, learning disabilities and ADHD are more common in children and adolescents who suffer from primary headaches [14,21,23]. 

In this current study, we found that children with learning difficulties were distributed similarly in the WH and MWH groups (48% and 52%, respectively) as opposed to children without learning difficulties, which were more prevalent in the WH group (48% vs. 21%). Children facing learning difficulties experience both midweek headaches and weekend headaches to an equal degree. This can be due to the pervasive impact of academic stress, which extends beyond specific moments, with the difficulty consistently present in the background. One could suggest that individuals without learning difficulties tend to finish their school assignments during the week, allowing them to have weekends free from academic responsibilities. In contrast, children facing learning difficulties may extend their school-related tasks and other duties into the weekend. This difference might contribute to the higher incidence of weekend headaches among children with learning difficulties. 

The main strength of this study is the prospective design, which uses a patient (or family)-filled diary filled for up to six weeks and is not based on a general retrospective history that is often subject to recall bias [4,26]. 

This study has some limitations: it consists of a relatively limited sample size originating from a single headache clinic. Additionally, a significant number of participants (40 out of 109) did not complete the headache diaries and were therefore excluded. The learning difficulties were self-reported, and other factors such as cervical pain, sitting postures, or other factors were not evaluated. Hopefully, these findings might encourage higher scale studies focusing on different patterns of headaches in children and eventually lead to better management of these patients. 

## 5. Conclusions

On the contrary to adults, children usually do not suffer from WH exclusively, but many suffer from both midweek and weekend headaches. Among those without learning difficulties, most headaches were on midweek days as opposed to those with learning difficulties. This indicates that learning challenges may play a role in the distribution of headache attacks throughout the week. To gain a more comprehensive understanding of the specific patterns, further research is essential. Exploring potential factors contributing to these occurrences will provide valuable insights into the complex interplay between learning difficulties and headache patterns, hopefully leading to more accurate management of headaches in children. 

## Figures and Tables

**Table 1:** Demographic characteristics of children with weekend headaches (WH) and children with midweek headaches (MWH).

	WH (n = 20)	MWH (n = 40)	Total	*p*-Value
Age, years, and mean	11.6	11.9	11.8	0.57
Primary school, no. (%)	13 (65%)	20 (50%)	33 (55%)	0.27
Junior High and High School, no. (%)	7 (35%)	20 (50%)	27 (45%)	
Male, no. (%)	9 (45%)	13 (32%)	22 (37%)	0.34
Female, no. (%)	11 (55%)	27 (68%)	38 (63%)	

**Table 2:** Lifestyle and stress triggers.

Triggers	WH (n = 20)	MWH (n = 40)	Total	*p*-Value
Academic stress, no. (%)	7 (35%)	19 (48%)	26 (43%)	0.36
Emotional stress, no. (%)	11 (55%)	22 (55%)	33 (55%)	1.00
Lack of sleep, no. (%)	11 (55%)	19 (48%)	30 (50%)	0.58
Excess screen hours, no. (%)	11 (55%)	13 (32%)	24 (40%)	0.09
Lack of physical activity, no. (%)	15 (75%)	23 (56%)	38 (63%)	0.18
Regular meals, no. (%)	8 (40%)	21 (53%)	29 (48%)	0.36

**Table 3:** Clinical characteristics of children with weekend headaches (WH) and children with midweek headaches (MWH).

	WH (n = 20)	MWH (n = 40)	Total	*p*-Value
Type of headache				0.90
Migraine, no. (%)	13 (65%)	23 (57%)	36 (60%)	0.58
Tension-type headache, no. (%)	3 (15%)	6 (15%)	9 (15%)	1.00
Mixed, no. (%)	3 (15%)	7 (17%)	10 (17%)	0.81
Undetermined, no. (%)	1 (5%)	4 (10%)	5 (8%)	0.51
Chronic headache, no. (%)	2 (10%)	11 (28%)	13 (22%)	0.12
Learning difficulties, no. (%)	13 (65%)	14 (35%)	27 (45%)	0.03

**Table 4:** Distribution of headaches during the week among children with primary headaches and learning difficulties and in children with primary headaches without learning difficulties.

	Primary Headache with Learning Difficulties n = 27 (45%)	Primary Headache without Learning Difficulties n = 33 (55%)	Total	*p*-Value
WH, no. (%)	13 (48%)	7 (21%)	20 (33%)	0.03
MWH, no. (%)	14 (52%)	26 (79%)	40 (67%)	

## Data Availability

Data are contained within the article.
